# lncRNA OSTN-AS1 May Represent a Novel Immune-Related Prognostic Marker for Triple-Negative Breast Cancer Based on Integrated Analysis of a ceRNA Network

**DOI:** 10.3389/fgene.2019.00850

**Published:** 2019-09-13

**Authors:** Zijian Liu, Mi Mi, Xiaoqian Li, Xin Zheng, Gang Wu, Liling Zhang

**Affiliations:** Cancer Center, Union Hospital, Tongji Medical College, Huazhong University of Science and Technology, Wuhan, China

**Keywords:** breast cancer, ceRNA network, immune infiltration, Gene Set Variation Analysis (GSVA), survival, bioinformatics

## Abstract

The competing endogenous RNA (ceRNA) networks are an effective method for investigating cancer; however, construction of ceRNA networks among different subtypes of breast cancer has not been previously performed. Based on analysis of differentially expressed RNAs between 150 triple-negative breast cancer (TNBC) tissues and 823 non–triple-negative breast cancer (nTNBC) tissues downloaded from TCGA database, a ceRNA network was constructed based on database comparisons using Cytoscape. Survival analysis and receiver operating characteristic curve data were combined to screen out prognostic candidate genes, which were subsequently analyzed using co-expressed functionally related analysis, Gene Set Variation Analysis (GSVA) pathway-related analysis, and immune infiltration and tumor mutational burden immune-related analysis. A total of 190 differentially expressed lncRNAs (DElncRNAs), 48 differentially expressed mRNAs (DEmRNAs), and 13 differentially expressed miRNAs (DEmiRNAs) were included in the ceRNA network between TNBC and nTNBC subtypes. Gene ontology analysis of mRNAs coexpressed with prognostic candidate lncRNAs (AC104472.1, PSORS1C3, DSCR9, OSTN-AS1, AC012074.1, AC005035.1, SIAH2-AS1, and ERVMER61-1) were utilized for functional prediction. Consequently, OSTN-AS1 was primarily related to immunologic function, for instance, immune cell infiltration and immune-related markers coexpression. The GSVA deviation degree was increased with OSTN increased expression. In addition, many important immune molecules, such as PDCD1 and CTLA-4, were strongly correlated in terms of their quantitative expression. Competing endogenous RNA networks may identify candidate therapeutic targets and potential prognostic biomarkers in breast cancer. In particular, OSTN-AS1 serves as a novel immune-related molecule and could be involved in immunotherapy efforts in the future.

## Introduction

Breast cancer is the most commonly diagnosed cancer among women worldwide (Bray et al., 2018). It is estimated that more than 250,000 patients were diagnosed with breast cancer in the US in 2017 (Waks and Winer, 2019). Based on the presence or absence of molecular markers for the estrogen receptor (ER), progesterone receptor (PR), and human epidermal growth factor 2 (ERBB2; formerly HER2), breast cancer is categorized into three major subtypes, including hormone receptor–positive/ERBB2-negative, ERBB2-positive, and triple-negative. Triple-negative breast cancer (TNBC) comprises approximately 15% of all breast tumors and is characterized by a lack of ER, PR, and ERBB2 expression, which conveys a higher risk of distant metastasis compared to the other two subtypes, collectively referred to as non–triple-negative breast cancer (nTNBC) (Foulkes et al., 2010). In addition to surgical treatment and radiation for local therapy, nTNBC can also be treated using hormone-dependent endocrine therapy or molecular-targeted therapy based on molecular marker to reduce the risk of recurrence and improve overall survival rate, while TNBC lacks this corresponding systemic therapy to increase treatment efficacy (Joensuu et al., 2006; Whelan et al., 2010; Early Breast Cancer Trialists’ Collaborative Group, 2011). Due to the obvious differences between TNBC and nTNBC in terms of risk of recurrence, metastasis, and treatment strategies, it is vital to explore differences in the molecular phenotypic regulatory networks between these two subtypes, which will provide not only novel potential therapeutic targets but also increased understanding of the different molecular mechanisms inherent to these different subtypes. With the development of molecular sequencing methodology, it has become highly efficient to explore correlations between molecular markers and prognosis among different tumor types.

Noncoding RNAs, including long noncoding RNA (lncRNA) and microRNA (miRNA), are an important class of pervasive genes involved in a variety of biological functions, particularly transcriptional regulation (Wang and Chang, 2011). High-throughput screening methods, such as next-generation sequencing, are advanced large-scale technologies used for genome-wide analysis with many advantages, such as low background noise, large dynamic range, and high reproducibility (Mortazavi et al., 2008; Nagalakshmi et al., 2010). Previous studies proposed a novel RNA mechanism, the competing endogenous RNA (ceRNA) hypothesis, comprising complex interactions between lncRNAs and miRNAs that influence the biological functions of mRNA at the posttranscriptional level. Using miRNA as a bridge, numerous lncRNAs can modulate expression of mRNAs within a ceRNA network (Ebert et al., 2007; Salmena et al., 2011; Xia et al., 2018). We can analyze differentially expressed RNAs, including lncRNAs, miRNAs, and mRNAs, to construct ceRNA networks for distinct subtypes of diseases and to explore potential markers associated with disease phenotype and prognosis.

In the current study, a ceRNA network containing 190 lncRNAs, 48 mRNAs, and 13 miRNAs was constructed based on differentially expressed RNAs between 150 TNBC and 823 nTNBC samples using sequencing data downloaded from TCGA database. After survival analysis and receiver operating characteristic (ROC) curve creation for each member in the network, we identified several prognostic markers, including eight lncRNAs and one mRNA. According to gene ontology (GO) analysis of coexpressed mRNAs that strongly correlated with candidate lncRNAs, we found that the biological functions of OSTN-AS1 were significantly associated with immunity and metabolism. Several immune-related genes involved in T- and B-cell receptor signaling pathways, such as PDCD1, CTLA-4, and CD79, yielded a strong linear relationship with expression of OSTN-AS1. OSTN-AS1 may act as a novel immune and prognostic marker in future research.

## Results

### Construction of a ceRNA Network in TNBC

**Figure 1:**
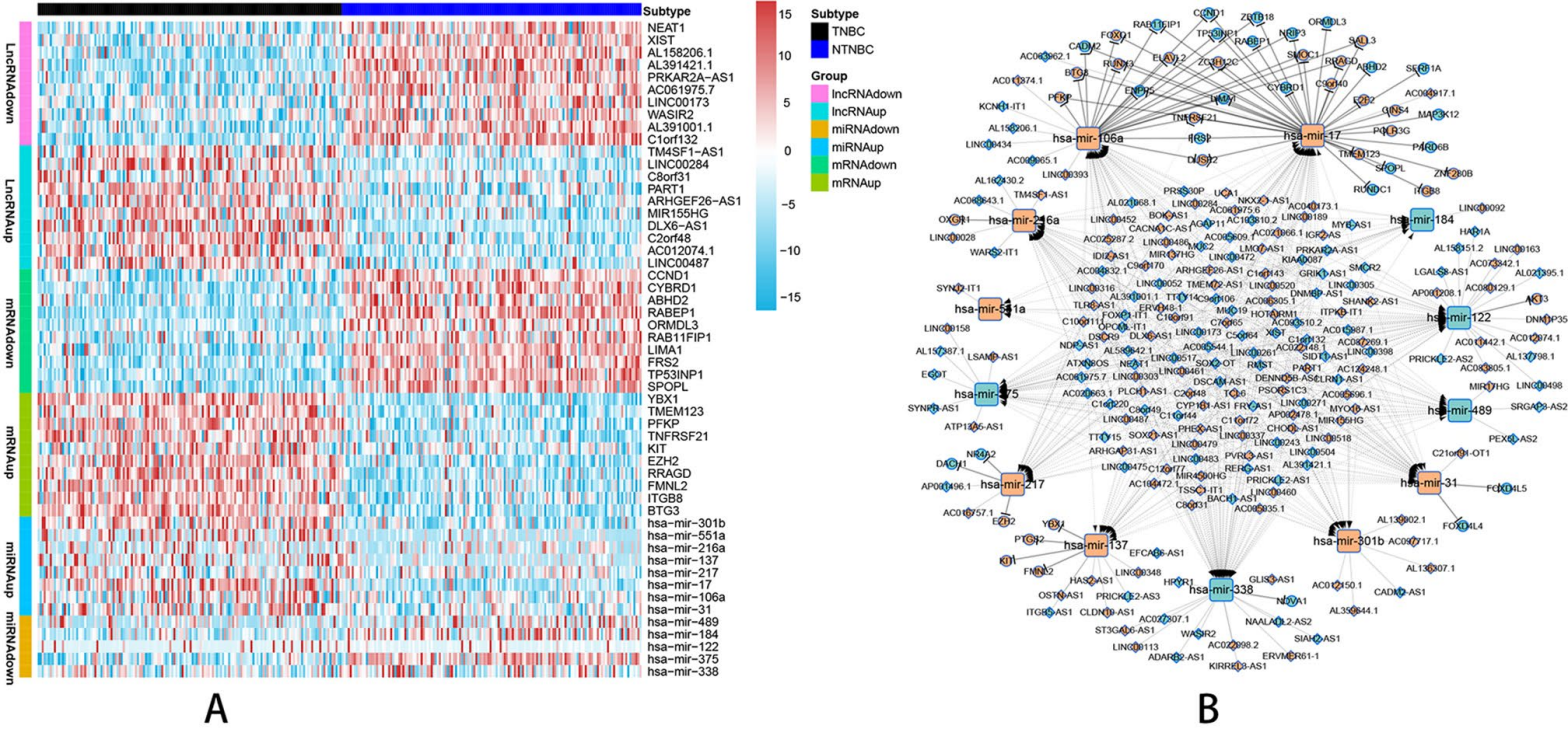
Visualization of the ceRNA network analyzed by differential screening and database comparison. **(A)** Heat map showing differences in gene expression between 150 TNBC samples and 150 nTNBC samples picked up randomly in 823 nTNBC samples by randomly selecting 10 RNAs from each RNA type except miRNAs. **(B)** CeRNA network containing 251 nodes and 598 edges in breast cancer demonstrated pair bonds among lncRNA–miRNA–mRNA. Diamonds, squares, and circles represent lncRNAs, miRNAs, and mRNAs, respectively. Light blue indicates upregulated genes in nTNBC subtype, and red–orange indicates upregulated genes in TNBC subtypes.

According to the probe annotation information from the Ensemble database, 19,676 mRNAs and 14,447 lncRNAs were separated from gene count expression matrix and downloaded from the Cancer Genome Atlas (TCGA) database. Differentially expressed RNAs were analyzed using the edgR package in R from 823 nTNBC samples and 150 TNBC samples with criteria of *P* < 0.01 and |log fold change [FC]| > 1. Next, 2,232 differentially expressed lncRNAs (1,249 TNBC upregulated and 983 TNBC downregulated vs. nTNBC samples), 3,764 differentially expressed mRNAs (2,131 TNBC upregulated and 1,633 TNBC downregulated vs. nTNBC samples), and 108 differentially expressed miRNAs (70 TNBC upregulated and 38 TNBC downregulated vs. nTNBC samples) were identified by comparing TNBC and nTNBC tissues. Among these differentially expressed RNAs, we constructed a ceRNA network containing two pairs of relationships, lncRNA-miRNA pairs and miRNA-mRNA pairs, which illustrate the complex competition and connection among endogenous RNAs. In total, 190 differentially expressed lncRNAs (DElncRNAs), 48 differentially expressed mRNAs (DEmRNAs) and 13 differentially expressed miRNAs (DEmiRNAs) were identified in the network (**Supplementary Table 1**), and the pairing relationships among these differentially expressed RNAs (DERNAs) were complicated but precise (**Supplementary Table 2**). Ten DERNAs, not including miRNAs, from each subgroup were randomly selected to construct a heat map to illustrate the differences in gene expression between TNBC and nTNBC (**Figure 1A**). Using Cytoscape software, we built ceRNA networks containing 251 nodes and 598 edges to clearly elucidate relationships among the different forms of RNAs (**Figure 1B**).

### Eight lncRNAs and One mRNA Were Identified as Prognostic Factors for TNBC

**Figure 2:**
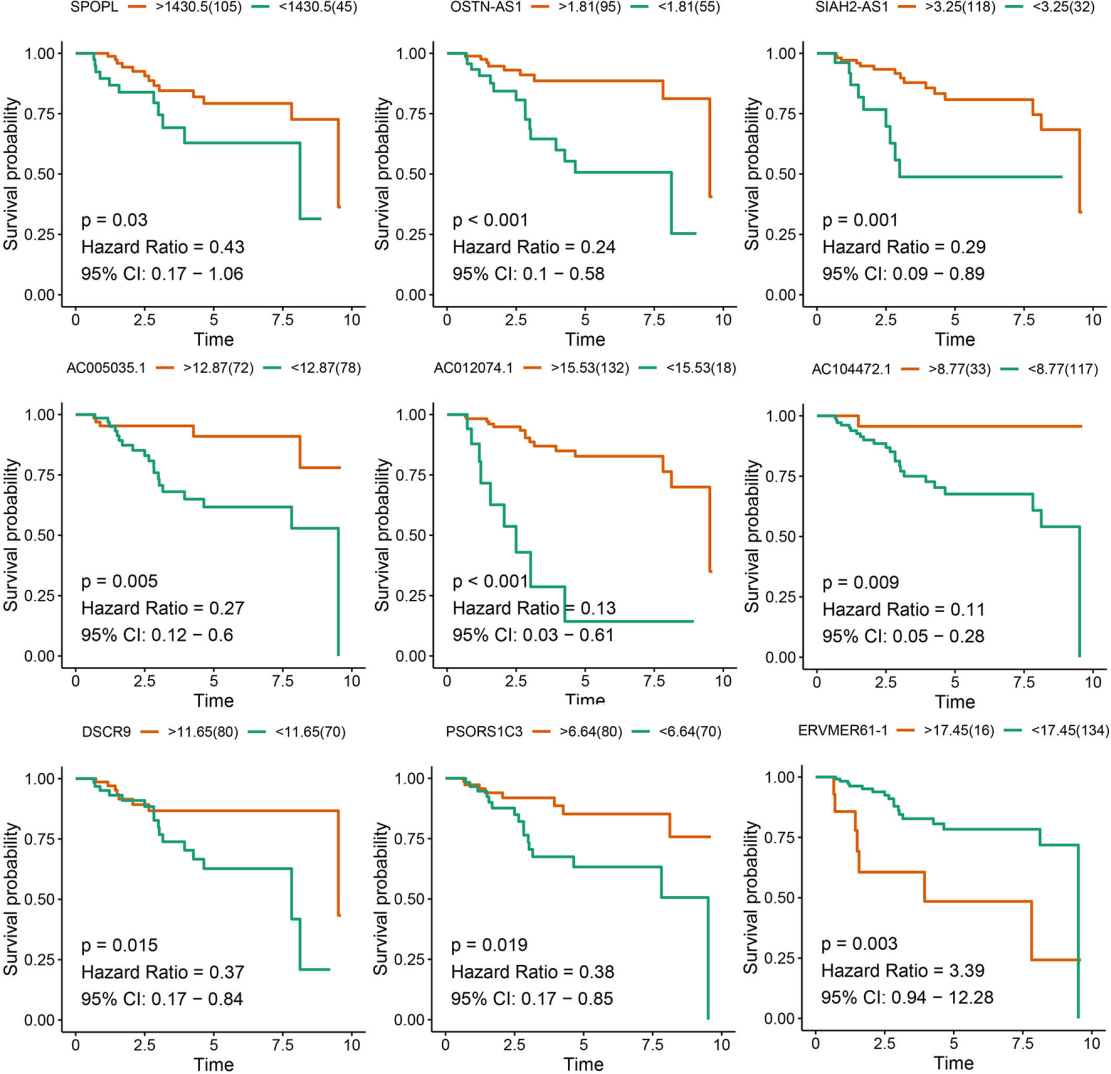
Analysis of prognostic RNAs screened from the ceRNA network. Overall survival analysis of 8 lncRNAs and mRNA SPOPL in TNBC.

After survival analysis and ROC curve analysis of each DERNA, eight lncRNAs (AC104472.1, PSORS1C3, DSCR9, OSTN-AS1, AC012074.1, AC005035.1, SIAH2-AS1, and ERVMER61-1) and one mRNA, SPOPL, were identified as prognostic factors in breast cancer. Receiver operating characteristic curves of all prognostic candidate genes are depicted together in **Supplementary Figure 1**. As shown in **Figure 2**, only ERVMER61-1 upregulated expression in TNBC was associated with unfavorable prognosis, whereas upregulated expression of other candidate genes (PSORS1C3, DSCR9, OSTN-AS1, SIAH2-AS1, AC104472.1, AC012074.1, AC005035.1, and SPOPL) was associated with favorable prognosis.

### OSTN-AS1 Is Potentially Associated With Immune Function

**Figure 3:**
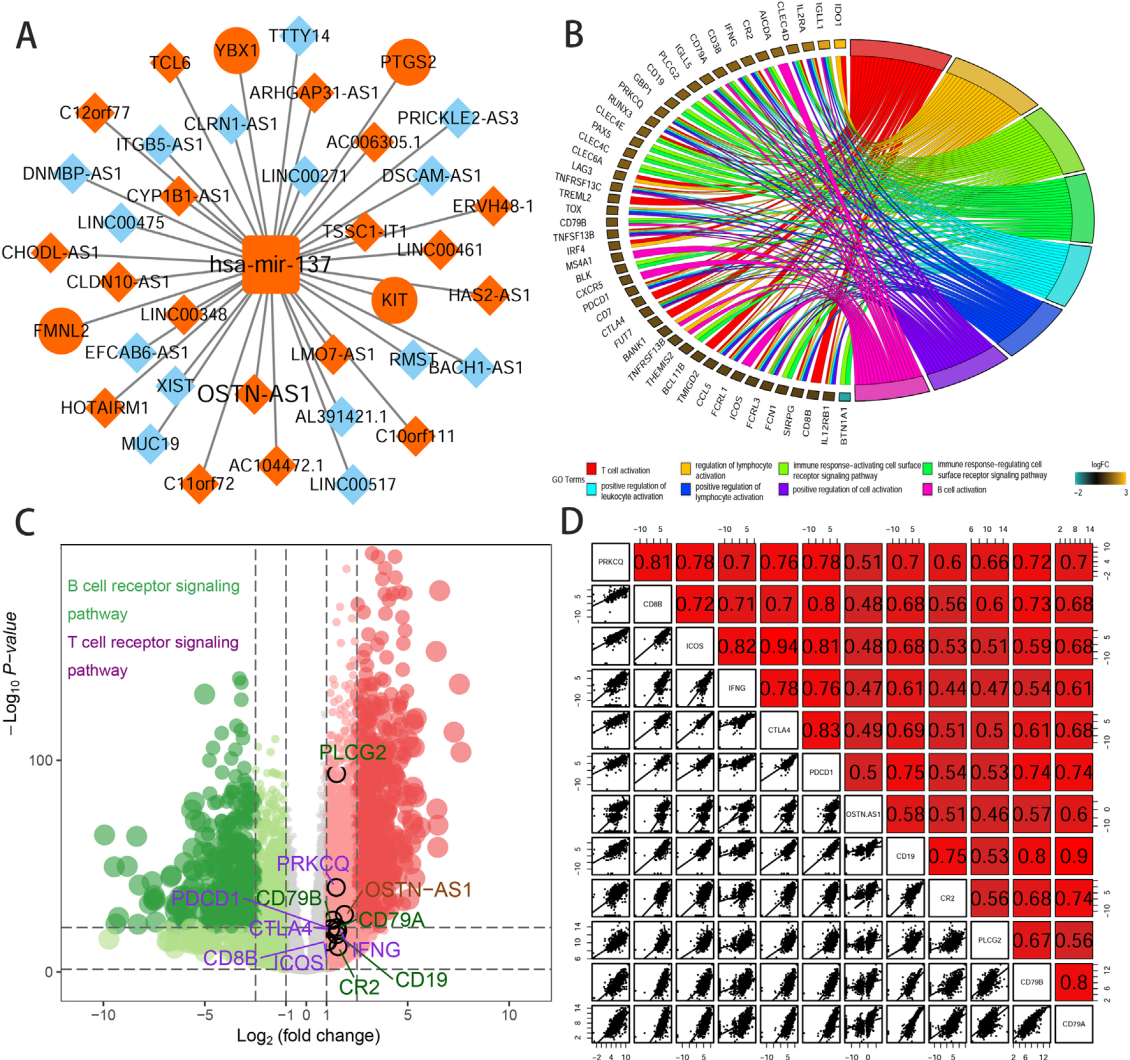
Predicted function of OSTN-AS1. **(A)** The Cytoscape map of OSTN-AS1 ceRNA network from the whole ceRNA network in BRCA. **(B)** The function of GO analysis of mRNAs coexpressed with OSTN-AS1. **(C)** Volcano plot of fold changes and –log10 (adjust *P* value) of differentially expressed RNAs, comprising lncRNAs, miRNAs, and mRNAs. Marked nodes are important pathways related to immunity that are involved in GO analysis of OSTN-AS1. **(D)** Correlation between OSTNAS1 and vital genes involved in T- and B-cell receptor signaling. Numbers indicate Pearson coefficients.

To further elucidate the biological function of the eight prognostic lncRNAs identified, we performed biological enrichment analysis using the database for annotation, visualization, and integrated discovery (DAVID) GO terms related to target genes. The results revealed only OSTN-AS1 and SIAH2-AS1, whose GO analysis of coexpressed mRNAs was significant among eight prognostic lncRNAs. The map of OSTN-AS1 ceRNA network from the whole ceRNA network in BRCA was separately shown in **Figure 3A**, and the specific predicted binding site of mir-137 located within the OSTN-AS1 was chr3:190931098 based on the information from Mircode database. Interestingly, OSTN-AS1 was found to be related to immune function (**Figure 3B**).

**Figure 4:**
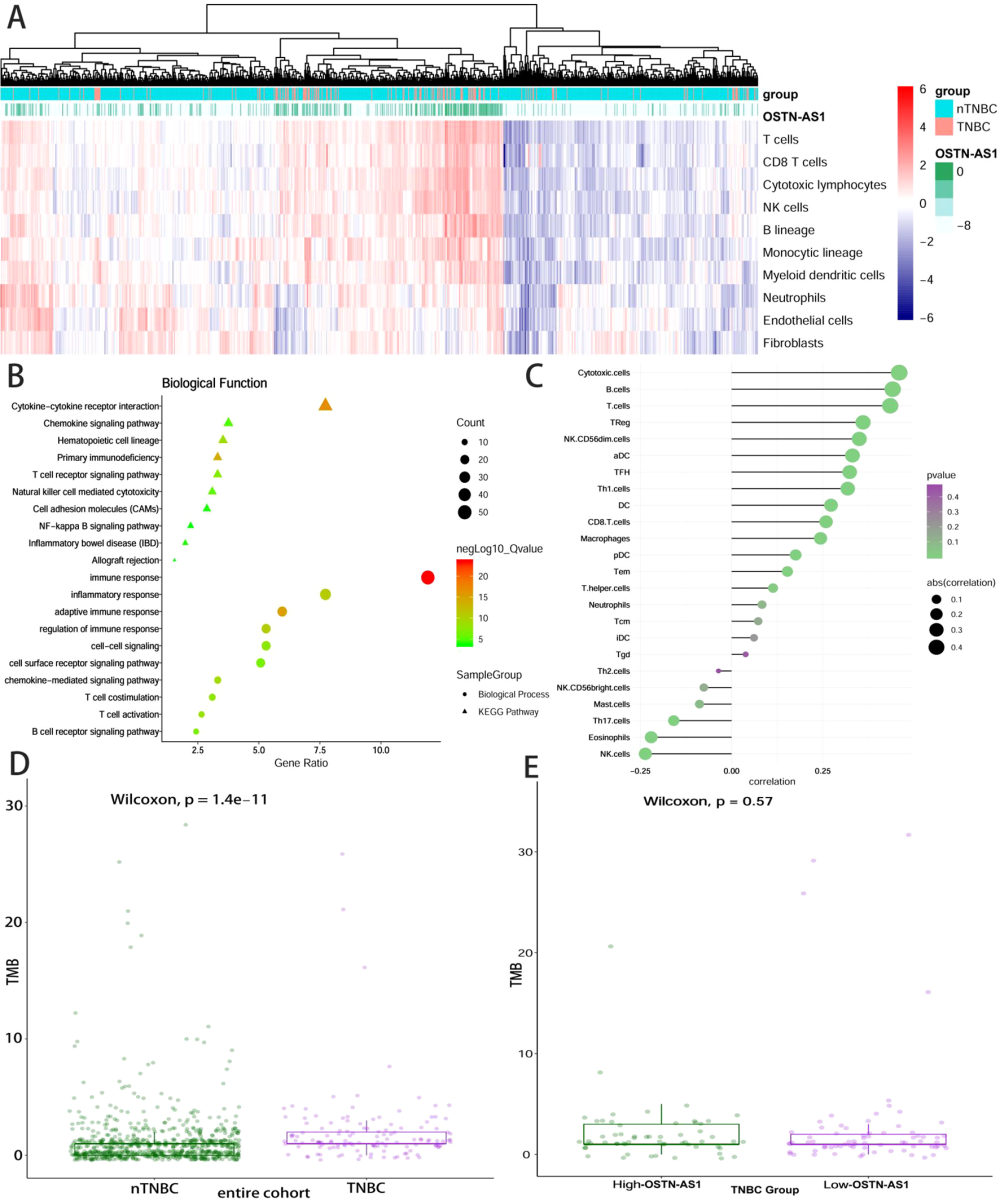
OSTN-AS1 was associated with immune response. **(A)** The MCP counter algorithm was utilized to analyze the degree of infiltration of immune cells with changing OSTN-AS1 expression. **(B)** Biological process and KEGG pathway analysis of differentially expressed genes between OSTN-AS1 high expression group and OSTN-AS1 low expression group. **(C)** The exhibition of correlation coefficient between the expression of OSTN-AS1 and several immune cell infiltration level calculated by ssGSEA analysis. **(D)** TMB levels of different subgroups in breast cancer. **(E)** TMB levels in TNBC according to the OSTN-AS1expression.

Some DEmRNAs, as mentioned previously, were strongly correlated with OSTN-AS1 and were enriched in T- and B-cell receptor signaling pathways, such as PDCD1, CTLA-4, CD79A, and CD79B (**Figure 3C**). Furthermore, these genes associated with immune function, including OSTN-AS1, were highly positively correlated with one another (**Figure 3D**). For further validation of OSTN-AS1 as a potential immune influencer, we calculated the degree of its immune infiltration *via* MCP counter in R to estimate the tendency of residential immune cells to vary with expression changes in OSTN-AS1 (**Figure 4A**). Although expression of OSTN-AS1 is relatively low in breast cancer, the tendency of its expression to be associated with immune infiltration was still significant. Additionally, using differentially expressed analysis about OSTN-AS1 high and low expression groups, the upregulated genes in OSTN-AS1 high expression group were mainly enriched in immune-related biological function and KEGG pathways (**Figure 4B**). Furthermore, ssGSEA analysis revealed that the expression of OSTN-AS1 was strongly positively correlated with several types of immune cell infiltration, such as cytotoxic cells, B cells, and T cells (**Figure 4C**). Tumor mutational burden (TMB) levels were relatively higher in the TNBC group compared to the nTNBC group. However, the difference of TMB between OSTN-AS1 high and low expression groups was not significant (**Figures 4D, E**).

### Expression of OSTN-AS1 Partially Reflects Pathway Differences *via* Gene Set Variation Analysis

**Figure 5:**
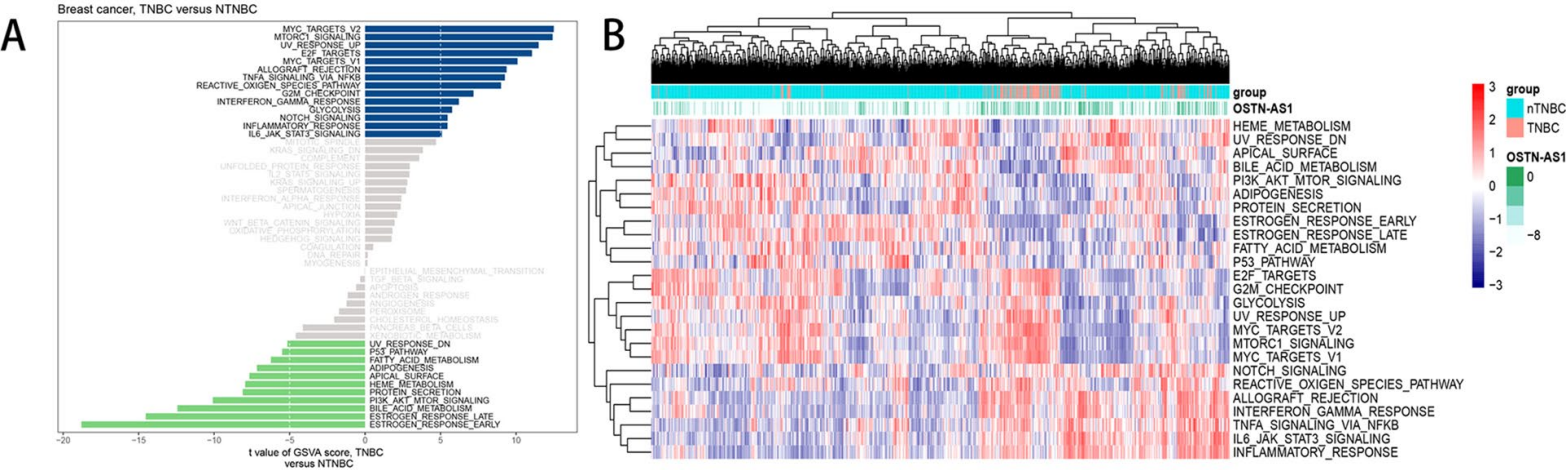
GSVA analysis displaying pathway differences. **(A)** Differences in pathway activities scored by GSVA between TNBC and nTNBC subtypes. *T* values are shown from a linear model. We set |*t*| > 5 as a cutoff value. The blue column indicates activated pathways in TNBC, and the green column indicates activated pathways in nTNBC. DN, down; UV, ultraviolet; v1, version 1; v2, version 2. **(B)** The variation tendency between increasing expression of OSTN-AS1 and the degree of differentially expressed pathways.

Analysis of hallmark pathway gene signatures highlighted that signaling pathways converging at various biological process were quite different between TNBC and nTNBC samples (**Figure 5A**). Of note, TNBC is more relevant in MYC-, NFκB-, NOTCH-, and E2F-related growth-promoting pathways, in agreement with the particularly aggressive and invasive properties of TNBC. In comparison, nTNBC is preferentially related to large hormonal fluctuations and metabolism, including estrogen response and other metabolic pathways. In addition, inflammatory and immune responses are also involved more so in TNBC than in nTNBC, as evidenced by MTORC1, IL-6, and inflammatory-related pathways. In different OSTN-AS1 expression groups, involved signaling pathways were different. High OSTN-AS1 expression was more related to immune associated pathways, for instance, interferon γ response pathway, IL-6–JAK–STAT3 signaling pathway, and allograft rejection pathway (**Figure 5B**). On the other hand, OSTN-AS1 might be connected with the gene sets involved in pathway assessment.

### OSTN-AS1 Expression Is Variable in Different Types of Cancers

**Figure 6:**
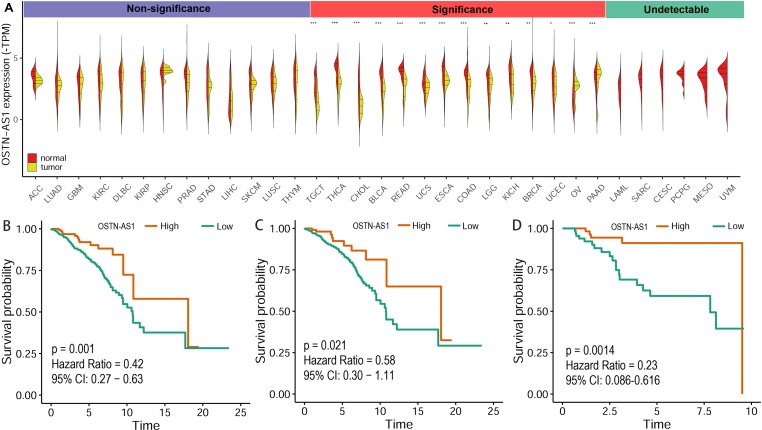
Prediction of OSTN-AS1 as a tumor suppressor. **(A)** Violin plot constructed using transcripts per million reads (TPM) data downloaded from TCGA tumor database and GTEx normal tissue database to illustrate expression of OSTN-AS1 between multiple cancers and their corresponding normal tissues. **(B-D)** Overall survival analysis of OSTNAS1 in BRCA, nTNBC and TNBC. The abbreviation for each tumor was put in the **Supplementary Table 3**.

The expression level of OSTN-AS1 in normal tissues and breast cancer as well as other types of cancer was analyzed. The results demonstrated that OSTN-AS1 expression was variable in different types of cancers (**Figure 6A**). In breast cancer, the expression of OSTN-AS1 was significantly lower than normal tissue. OSTN-AS1 low expression was associated with a poor prognosis in patients with BRCA, nTNBC, and TNBC (**Figures 6B–D**). As shown in **Figure 6A**, expression of OSTN-AS1 was significantly downregulated in 14 types of cancers, while in other types of cancers (adrenocortical carcinoma, lung adenocarcinoma, glioblastoma multiforme, etc.), the difference between normal tissues and cancers was not significant. In six types of cancers (acute myeloid leukemia, sarcoma, cervical squamous cell carcinoma, pheochromocytoma and paraganglioma, mesothelioma, and uveal melanoma), OSTN-AS1 was not detected. The function of OSTN-AS1 in tumorigenesis needs to be further explored.

## Discussion

Previous studies have revealed that many noncoding RNAs, including differentially expressed lncRNAs and miRNAs, modulate the expression of many mRNAs at multiple levels, such as through transcriptional regulation, posttranscriptional regulation, and epigenetic modification, and are considered tumor suppressor genes and novel oncogenes involved in the progression and metastasis of various cancers (Kurokawa et al., 2009; Wang et al., 2011; Guttman and Rinn, 2012; Liu et al., 2017). Since the characteristics of miRNAs have been largely elucidated and their mode of action has been generalized and summarized in detail (Dragomir et al., 2018), lncRNAs were shown as potential biomarkers correlated with tumor progression and metastasis and have significant advantages as prognostic and diagnostic molecular targets (Augoff et al., 2012; Rönnau et al., 2014; Huang et al., 2018). In particular, lncRNAs, once viewed as garbage, account for an important proportion of the human genome and have significant potential in biomedical research based on the ceRNA theory that the biological effects of mRNAs are modulated directly by lncRNAs or indirectly by competitive binding to miRNA response elements (Cesana et al., 2011; Karreth et al., 2011; Tay et al., 2011).

In the current study, we constructed a ceRNA network containing 190 lncRNAs, 48 mRNAs, and 13 miRNAs and analyzed differentially expressed pathways to identify distinctions at the transcriptional level between two different tumor subtypes in terms of prognosis, molecular signature, and treatment. Pathway analysis by Gene Set Variation Analysis (GSVA) in our study was distinct from GSEA analysis or GO analysis of differentially expressed genes, as GSVA provides a more accurate definition of the pathways by which genes are classified. From pathway differences, we found that the malignant growth ability of TNBC was stronger than that of nTNBC. For example, pathway enrichment for cell proliferation and tumor stem cells, including genes such as MYC, NFκB, and WNT, was more relevant in TNBC than in nTNBC subtypes. c-MYC is a crucial oncogenic transcription factor that is highly expressed in basal-like breast cancer (Chen and Olopade, 2008), and Notch signaling, an evolutionarily conserved cell fate determination pathway, is involved in multiple aspects of tumor biology, from angiogenesis to cancer stem cell maintenance to tumor immunity (Espinoza and Miele, 2013). From our results, differences in pathways in nTNBC were primarily involved in hormone- and metabolism- associated pathways. We found that many differentially expressed genes present in the GSVA gene set included some of the genes that are in the network contributing to gene sets in GSVA analysis. POLR3G upregulation in TNBC is contained in MTORC1 signaling, RABEP1 upregulation in nTNBC is included in ER response late signaling, and GINS4 upregulation in TNBC is involved in E2F signaling. The function of these three mRNAs in our network has not been previously reported in breast cancer, indicating that mRNAs involved in both networks or gene sets in GSVA have the potential to be further investigated for deeper analysis between two different groups in BRCA.

As far as we know, the function of our prognostic genes is mostly unknown. Through coexpression analysis of prognostic lncRNAs, we employed GO analysis of the mRNAs correlated with lncRNAs, and only OSTN-AS1 and SIAH2-AS1 were biologically significant. SIAH2-AS1 was upregulated in nTNBC samples and was correlated with estrogen signaling and metabolism-related pathways (**Supplementary Figure 2**), in agreement with GSVA results. OSTN-AS1 was primarily concentrated in immune-related pathways, and we found it was positively correlated with the degree of immune cell infiltration of T/B cells and natural killer cells, indicating it might participate in immune regulation and identify new immunotherapy monitoring points. With its strong correlation with genes, such as PDCD1, CTLA-4, CD79 involved in T-/B-cell receptor signaling, and increased TMB levels, we firmly believe that OSTN-AS1 is related to the immune process and that it has potential as a novel immune-related marker. The GTEx database makes up for the defect of fewer normal samples in TCGA database, and OSTN-AS1 was downregulated in most tumors versus normal tissues. In light of these findings, OSTN-AS1 may represent a novel immune-related prognosis.

In summary, in the current study, to our knowledge, this is the first ceRNA network between TNBC and nTNBC subtypes in BRCA based on lncRNA–miRNA–mRNA pair bonds. Prognostic genes based on diagnosis and prognosis were screened out as candidate genes with the significance of further analysis in the network, and the predicted function of these genes requires additional validation. There are some limitations to this study, including there is no suitable dataset containing large amounts of noncoding RNA data to verify our findings. On the other hand, the relationships between the pairs of lncRNAs and miRNAs were complicated so that we only briefly analyzed the relationship between several pairs of miRNA and mRNA, and the complex regulatory networks involved remain to be further elucidated. In terms of survival analysis, the univariate Cox model is primarily adopted. Since grouping and verification of multivariate Cox models are complex, we will further verify these findings using a multivariate Cox model, which was not mentioned in this article. All of these analyses were obtained by data mining based on an algorithm model and lacked rigorous experimental demonstration. In subsequent studies, our experimental research will further demonstrate and improve our findings concerning this ceRNA network in breast cancer.

## Methods and Materials

### Data Collection and Screening

Using the Genomic Data Commons (GDC) data portal officially provided by The Cancer Genome Atlas (TCGA, http://tcga-data.nci.nih.gov/tcga/) database, high-throughput sequencing-counts data (HTSeqcounts) of Transcriptome Profiling containing mRNA, lncRNA, and miRNA of BRCA were downloaded online for further sample filtering. Clinical information from BRCA patients was also obtained from TCGA and served as a condition for sample selection. Eligible sample screening conditions were as follows: (1) samples have both transcriptome expression data and clinical prognostic information; (2) samples excised from primary tumors were selected according to the naming principle of TCGA samples; (3) samples have a clear ER, PR, and ERBB2 status, and immunohistochemistry and *in situ* hybridization results of ERBB2 in the TNBC samples are consistent. After this rigorous screening, a total of 973 BRCA samples were included, comprising 823 nTNBC samples and 150 TNBC samples. According to the result of survival analysis of OSTN-AS1, TPM of 3.07 was chosen as a cutoff to divide patients into OSTN-AS1 high expression and low expression groups.

### Differentially Expressed Gene Analysis

Expression matrices of lncRNAs and mRNAs were separated according to the Ensemble database for human gene ID annotation information, and the annotation of miRNAs was ready-made with downloaded expression profiles. Differentially expressed RNAs between TNBC and nTNBC were analyzed using the edgeR package in R with a cutoff of false discovery rate-adjusted *P* < 0.01 and |logFC| > 1 to identify potential molecules during the development of two subtypes of BRCA. The heat map and volcano plot to illustrate differentially expressed RNAs were plotted using R script.

### Construction of the ceRNA Network

Using the base complementary pairing principle and experimental verification, some authoritative databases identified relationship pairs, such as lncRNA-miRNA and miRNA-mRNA, which are effective for constructing a ceRNA network centered on miRNA. Briefly, differentially expressed lncRNAs were first utilized to identify target miRNAs using the miRcode database, and then successfully paired miRNAs 3p or 5p transcript information was obtained by comparison within the starBase database. Next, three databases, TargetScan (http://www.targetscan.org/), miRTarBase (http://mirtarbase.mbc.nctu.edu.tw/), and miRDB (http://www.mirdb.org/), were used to predict corresponding target mRNAs of miRNAs. Only paired lncRNAs-miRNAs and mRNAs predicted by all three databases were regarded as DERNAs, resulting in identification of 190 lncRNAs, 13 DEmiRNAs, and 48 DEmRNAs. Finally, DERNAs satisfying the above matching conditions were selected to construct the ceRNA network using Cytoscape visualization software.

### Gene Function Enrichment Analysis

The Database for Annotation, Visualization, and Integrated Discovery (DAVID) (http://david.abcc.ncifcrf.gov/) was utilized to perform functional enrichment analysis including GO terms composed of biological process, molecular function, and cellular component among DEmRNAs and coexpressed genes of lncRNAs. The ClusterProfiler package in R was also engaged in functional exhibition of coexpressed genes of DElncRNAs. A *P* < 0.05 was considered to represent significant enrichment, while transcription factor screening criterion was a *P* value of <0.01.

### Gene Set Variation Analysis

In case of overlapping pathways and pathway redundancies, each gene set associated with a hallmark pathway predominantly present in the molecular signature database and other described curated datasets was trimmed to a collection of unduplicated genes, and all genes linked to two or more pathways retaining greater than 70% of their associated genes in most gene sets were removed (Lambrechts et al., 2018). To reveal pathway enrichment between samples from different groups, we used the GSVA_1.30.0 package in R (Hänzelmann et al., 2013) to evaluate *t* score and assign pathway activity conditions and ggplot2_3.1.0 in R to display distinctions in pathway activation between the TNBC and nTNBC groups.

### ROC Curve and Survival Analysis of DERNAs in the ceRNA Network

Univariate Cox proportional hazards regression analysis and ROC curve analyses of DERNAs containing DElncRNAs, DEmiRNAs, and DEmRNAs in ceRNA network were performed to identify prognosis-related RNAs for further mining. Survival analyses of DERNAs were conducted for TNBC, nTNBC, and the entire cohort with a cutoff of *P* < 0.01. Receiver operating characteristic curve assessed the diagnostic efficiency of DERNAs in the two subgroups with a criterion of area under curve >0.7. When an RNA simultaneously satisfied these two conditions, the *P* value can be relaxed to 0.05 if needed and can be regarded as a key prognostic signature for deeper analysis. Both analyses were conducted and depicted using R.

### Analysis of Functional Prediction of Prognostic DElncRNAs

Given that the function of most prognostic DElncRNAs is undiscovered, we utilized the coexpression linear regression method to identify mRNAs with known functions related to lncRNAs, which can be helpful in predicting possible function of lncRNAs. We evaluated the degree of infiltration of immune cells by changing levels of a particular gene using the MCPcounter_1.1.0 package in R to establish the potential relationship between the gene of interest and immunotherapy (Becht et al., 2016). We used the somatic called variants as determined by TCGA as the raw mutation count and 35 Mb as the estimate of exome size. Tumor mutation burden was calculated in R, and the Wilcoxon test was used to perform statistical tests. The Genotype-Tissue Expression database provided expression levels in normal tissue to validate the expression of candidate genes between cancer samples and normal tissues, and the TPM value processed by TOIL (Vivian et al., 2017), downloaded from UCSC xena (https://xenabrowser.net/datapages/), was acquired for MCP counter and violin plot that show differences in expression of a gene between multiple cancer types and their corresponding normal tissues. The infiltration levels of immune cell types in OSTN-AS1 high and low expression groups were quantified by ssGSEA in R package gsva (Bindea et al., 2013).

## Conclusions

We constructed a ceRNA network in TNBC, and eight lncRNAs and one mRNA were identified as associated with TNBC prognosis. OSTN-AS1 represents a novel immune-related molecule, and its overexpression is associated with favorable prognosis in TNBC. OSTN-AS1 may represent a novel immune-related prognostic marker, and its application in immunotherapy needs to be further studied.

## Data Availability

Publicly available datasets were analyzed in this study. The data can be found in the TCGA database: https://portal.gdc.cancer.gov/.

## Author Contributions

GW and LZ designed the experiments. ZL, MM, and XL collected the data. ZL and XZ analyzed the data. ZL and LZ wrote the manuscript. All authors approved the final manuscript.

## Funding

The study was supported by grant from the National Natural Science Foundation of China (no. 81672940) and the Clinical Research Physician Program of Tongji Medical College, Huazhong University of Science and Technology (no. 5001530053).

## Conflict of Interest Statement

The authors declare that the research was conducted in the absence of any commercial or financial relationships that could be construed as a potential conflict of interest.

## Abbreviations

TNBC, triple-negative breast cancer; nTNBC, non–triple–negative breast cancer; lncRNA, long noncoding RNA; ceRNA, competing endogenous RNA; ROC, receiver operating characteristic; AUC, area under curve; OS, overall survival; GO, gene ontology; TMB, tumor mutational burden; GDC, Genomic Data Commons; TCGA, The Cancer Genome Atlas; FC, fold change; DAVID, The Database for annotation, Visualization, and Integrated Discovery; GSVA, Gene Set Variation Analysis; GTEx, Genotype-Tissue Expression.
