# Supplementary material for: lncRNA OSTN-AS1 May Represent a Novel Immune-Related Prognostic Marker for Triple-Negative Breast Cancer Based on Integrated Analysis of a ceRNA Network
Source: Front Genet. 2019 Sep 13;10:850. doi: 10.3389/fgene.2019.00850 (PMC6753250; doi:10.3389/fgene.2019.00850)
Supplement: Supplementary file 1 [file Table_1.docx]

|  | Up-regulated in TNBC | Down-regulated in nTNBC |
| --- | --- | --- |
| LncRNA | IGF2AS,PART1,C2orf48,AC021066.1,C10orf111,AC087269.1,LINC00303,C8orf31,AP002478.1,AC004917.1,AC012074.1,C10orf91,AC022148.1,LINC00518,C11orf72,LINC00158,TCL6,AC061975.6,C9orf170,PSORS1C3,LINC00487,DNM1P35,AC006305.1,UCA1,AC104472.1,MIR17HG,LINC00189,C7orf65,AC012150.1,CLDN10-AS1,PHEX-AS1,TMEM72-AS1,LINC00393,  TSSC1-IT1,LINC00337,LINC00092,LINC00113, DNM1P35, C12orf77,SHANK2-AS1,LINC00348,  SOX21-AS1,C1orf143,ITPKB-IT1,LMO7-AS1,MIR4500HG,AC022098.2,LINC00452,AC016757.1,DSCR9,ERVMER61-1,LINC00486,MIR137HG,  CHODL-AS1,DLX6-AS1,IDI2-AS1,CYP1B1-AS1,  ERVH48-1,OSTN-AS1,TLR8-AS1,LINC00028,  AC005035.1,HOTAIRM1,SYNJ2-IT1,LINC00460,  LINC00284,BOK-AS1,LINC00163,MIR155HG,  MYO16-AS1,LINC00479,ATP13A5-AS1,GLIS3-AS1,  LINC00316,AP001208.1,AC073342.1, PLCH1-AS1  ST3GAL6-AS1,AL136307.1,,AC011374.1,AC005696.1  AC025287.2,AC080129.1,AL359644.1,AC097717.1,TM4SF1-AS1,C21orf91OT1,LSAMP-AS1,ARHGAP31-AS1  ,AC124248.1,AC011442.1,PVRL3-AS1,AC083805.1,  AL139002.1,ARHGEF26-AS1,LINC00461,CACNA1C-AS1,HAS2-AS1,NKX2-1-AS1,DENND5B-AS1,AC040173.1,KIRREL3-AS1,LINC00520 | KIAA0087,AGAP11,LINC00483,AC009065.1,PRSS30P,  GRIK1-AS1,C11orf44,TTTY14,C5orf64,C9orf106,  LINC00305,LINC00173,MUC2,AC061975.7,AC004832.1,  C1orf132,AL391421.1,MUC19,ADARB2-AS1,C1orf220,  AL021068.1,AC005544.1,LINC00243,AC103810.2,  AL158151.2,AC027307.1,LGALS8-AS1,EFCAB6-AS1,  SMCR2,WARS2-IT1,PRKAR2A-AS1,LINC00475,HAR1A,  PRICKLE2-AS3,NAALADL2-AS2,LINC00434,DNMBP-AS1,  AP001496.1,SRGAP3-AS2,XIST,ATXN8OS,WASIR2,  BACH1-AS1,LINC00271,LINC00472,TTTY15,KCNH1-IT1,  DSCAM-AS1,EGOT,NDP-AS1,AC015987.1,MYB-AS1,  FRY-AS1,AL137798.1,LINC00398,AC068643.1,  AL162430.2,CLRN1-AS1,SIDT1-AS1,CADM2-AS1,  AL158206.1,AL021395.1,PRICKLE2-AS2,SYNPR-AS1,  PRICKLE2-AS1,FOXP1-IT1,AL391001.1,SOX2-OT,  AC063962.1,SIAH2-AS1,ITGB5-AS1,PEX5L-AS2,  AL157387.1,NEAT1,LINC00504,LINC00498,AC005609.1,  HPYR1,OPCML-IT1,C8orf49,RERG-AS1,RMST,  AL589642.1,AC020663.1,LINC00517,AC093510.2,  LINC00052,LINC00261 |
| mRNA | PTGS2,SMOC1,OXGR1,ZNF280B,FOXQ1,ELAVL2  PFKP,SALL3,RUNX3,BTG3,POLR3G,E2F2,ITGB8,  RRAGD,TNFRSF21,YBX1,FMNL2,C9orf40,GINS4,  EZH2,ZC3H12C,TMEM123,KIT,AKT3,DUSP2 | ZBTB18,LIMA1,SERF1A,NR4A2,RUNDC1,SPOPL,MAP3K12,  FRS2,ABHD2,RAB11FIP1,TP53INP1,FOXD4L5,ORMDL3,  RABEP1,CYBRD1,CCND1,ENPP5,FOXD4L4,CADM2,  PARD6B,NRIP3,DACH1,NOVA1 |
| miRNA | hsa-mir-17,hsa-mir-217,hsa-mir-551a,hsa-mir-106a,  hsa-mir-301b,hsa-mir-31,hsa-mir-216a,hsa-mir-137 | hsa-mir-122,hsa-mir-338,hsa-mir-375,  hsa-mir-184,hsa-mir-489 |

**Supplement Table 1.** Differentially expressed genes in ceRNA network.
