# Supplementary material for: lncRNA OSTN-AS1 May Represent a Novel Immune-Related Prognostic Marker for Triple-Negative Breast Cancer Based on Integrated Analysis of a ceRNA Network
Source: Front Genet. 2019 Sep 13;10:850. doi: 10.3389/fgene.2019.00850 (PMC6753250; doi:10.3389/fgene.2019.00850)
Supplement: Supplementary file 2 [file Table_2.docx]

**Supplement Table 2.** Paired lncRNA-miRNA and miRNA-mRNA in ceRNA network.

| Name1 | Name2 |
| --- | --- |
| XIST | hsa-mir-301b,hsa-mir-106a,hsa-mir-137,-+hsa-mir-17,hsa-mir-216a,hsa-mir-217,  hsa-mir-122,hsa-mir-31,hsa-mir-338,hsa-mir-375,hsa-mir-489, |
| WASIR2 | hsa-mir-338 |
| WARS2-IT1 | hsa-mir-216a |
| UCA1 | hsa-mir-184,hsa-mir-122 |
| TTTY15 | hsa-mir-106a,hsa-mir-17,hsa-mir-216a,hsa-mir-122,hsa-mir-31,hsa-mir-489, |
| TTTY14 | hsa-mir-106a,hsa-mir-137,hsa-mir-17,hsa-mir-184,hsa-mir-217,hsa-mir-31,hsa-mir-338, |
| TSSC1-IT1 | hsa-mir-137,hsa-mir-216a, |
| TMEM72-AS1 | hsa-mir-301b,hsa-mir-184,hsa-mir-216a,hsa-mir-338,hsa-mir-489, |
| TM4SF1-AS1 | hsa-mir-216a, |
| TLR8-AS1 | hsa-mir-31,hsa-mir-338, |
| TCL6 | hsa-mir-301b,hsa-mir-106a,hsa-mir-137,hsa-mir-17,hsa-mir-216a,hsa-mir-217,  hsa-mir-122,hsa-mir-31,hsa-mir-338,hsa-mir-375,hsa-mir-489, |
| SYNPR-AS1 | hsa-mir-375, |
| SYNJ2-IT1 | hsa-mir-551a, |
| ST3GAL6-AS1 | hsa-mir-338, |
| SRGAP3-AS2 | hsa-mir-489, |
| SOX2-OT | hsa-mir-301b,hsa-mir-122,hsa-mir-31,hsa-mir-338,hsa-mir-375, |
| SOX21-AS1 | hsa-mir-301b,hsa-mir-338,hsa-mir-489, |
| SMCR2 | hsa-mir-31,hsa-mir-338, |
| SIDT1-AS1 | hsa-mir-106a,hsa-mir-17,hsa-mir-31,hsa-mir-338, |
| SHANK2-AS1 | hsa-mir-106a,hsa-mir-17,hsa-mir-338, |
| RMST | hsa-mir-301b,hsa-mir-137,hsa-mir-17,hsa-mir-31,hsa-mir-338,hsa-mir-375,hsa-mir-489, |
| RERG-AS1 | hsa-mir-216a,hsa-mir-31, |
| PVRL3-AS1 | hsa-mir-106a,hsa-mir-17,hsa-mir-31,hsa-mir-338, |
| PSORS1C3 | hsa-mir-551a,hsa-mir-301b,hsa-mir-216a, |
| PRSS30P | hsa-mir-184,hsa-mir-216a,hsa-mir-122, |
| PRKAR2A-AS1 | hsa-mir-551a,hsa-mir-106a,hsa-mir-217,hsa-mir-338, |
| PRICKLE2-AS3 | hsa-mir-137, |
| PRICKLE2-AS2 | hsa-mir-122, |
| PRICKLE2-AS1 | hsa-mir-106a,hsa-mir-17, |
| PLCH1-AS1 | hsa-mir-106a,hsa-mir-217,hsa-mir-489, |
| PHEX-AS1 | hsa-mir-301b,hsa-mir-216a,hsa-mir-122,hsa-mir-31,hsa-mir-338, |
| PEX5L-AS2 | hsa-mir-489, |
| PART1 | hsa-mir-301b,hsa-mir-122,hsa-mir-31, |
| OSTN-AS1 | hsa-mir-137, |
| OPCML-IT1 | hsa-mir-106a,hsa-mir-17,hsa-mir-184,hsa-mir-375, |
| NKX2-1-AS1 | hsa-mir-301b,hsa-mir-17,hsa-mir-216a,hsa-mir-31, |
| NEAT1 | hsa-mir-301b,hsa-mir-106a,hsa-mir-17,hsa-mir-216a,hsa-mir-217,hsa-mir-122,hsa-mir-31,  hsa-mir-338,hsa-mir-489, |
| NDP-AS1 | hsa-mir-106a,hsa-mir-122, |
| NAALADL2-AS2 | hsa-mir-338, |
| MYO16-AS1 | hsa-mir-338,hsa-mir-489, |
| MYB-AS1 | hsa-mir-301b,hsa-mir-217, |
| MUC2 | hsa-mir-106a,hsa-mir-184,hsa-mir-122,hsa-mir-338, |
| MUC19 | hsa-mir-551a,hsa-mir-301b,hsa-mir-106a,hsa-mir-137,hsa-mir-17,hsa-mir-184,hsa-mir-216a,  hsa-mir-217,hsa-mir-122,hsa-mir-31,hsa-mir-338,hsa-mir-375,hsa-mir-489, |
| MIR4500HG | hsa-mir-301b,hsa-mir-216a,hsa-mir-31, |
| MIR17HG | hsa-mir-489, |
| MIR155HG | hsa-mir-217,hsa-mir-338,hsa-mir-375, |
| MIR137HG | hsa-mir-217,hsa-mir-31,hsa-mir-338, |
| LSAMP-AS1 | hsa-mir-375, |
| LMO7-AS1 | hsa-mir-106a,hsa-mir-137,hsa-mir-17,hsa-mir-122,hsa-mir-375,hsa-mir-489, |
| LINC00520 | hsa-mir-106a,hsa-mir-17,hsa-mir-217,hsa-mir-31,hsa-mir-375, |
| LINC00518 | hsa-mir-216a,hsa-mir-375, |
| LINC00517 | hsa-mir-106a,hsa-mir-137,hsa-mir-17,hsa-mir-216a,hsa-mir-122,hsa-mir-31,hsa-mir-338, |
| LINC00504 | hsa-mir-338,hsa-mir-375, |
| LINC00498 | hsa-mir-122, |
| LINC00487 | hsa-mir-106a,hsa-mir-216a,hsa-mir-31,hsa-mir-338, |
| LINC00486 | hsa-mir-122,hsa-mir-31,hsa-mir-338, |
| LINC00483 | hsa-mir-106a,hsa-mir-17,hsa-mir-216a,hsa-mir-122,hsa-mir-31, |
| LINC00479 | hsa-mir-216a,hsa-mir-217,hsa-mir-375, |
| LINC00475 | hsa-mir-137,hsa-mir-489, |
| LINC00472 | hsa-mir-106a,hsa-mir-184,hsa-mir-489, |
| LINC00461 | hsa-mir-106a,hsa-mir-137,hsa-mir-216a,hsa-mir-122,hsa-mir-31,hsa-mir-338,hsa-mir-489, |
| LINC00460 | hsa-mir-338,hsa-mir-489, |
| LINC00452 | hsa-mir-17,hsa-mir-122,hsa-mir-338, |
| LINC00434 | hsa-mir-106a, |
| LINC00398 | hsa-mir-338,hsa-mir-375, |
| LINC00393 | hsa-mir-106a, |
| LINC00348 | hsa-mir-137, |
| LINC00337 | hsa-mir-106a,hsa-mir-17,hsa-mir-216a,hsa-mir-217,hsa-mir-338,hsa-mir-375, |
| LINC00316 | hsa-mir-122, hsa-mir-338, |
| LINC00305 | hsa-mir-216a, hsa-mir-338, |
| LINC00303 | hsa-mir-216a, hsa-mir-122, |
| LINC00284 | hsa-mir-338, |
| LINC00271 | hsa-mir-137, hsa-mir-216a, hsa-mir-31, |
| LINC00261 | hsa-mir-301b,hsa-mir-31,hsa-mir-338,hsa-mir-375, |
| LINC00243 | hsa-mir-106a,hsa-mir-17,hsa-mir-122,hsa-mir-338,hsa-mir-375, |
| LINC00189 | hsa-mir-216a, hsa-mir-217, |
| LINC00173 | hsa-mir-301b,hsa-mir-17,hsa-mir-338,hsa-mir-375, |
| LINC00163 | hsa-mir-122, |
| LINC00158 | hsa-mir-375, |
| LINC00113 | hsa-mir-338, |
| LINC00092 | hsa-mir-184,hsa-mir-17,hsa-mir-217,hsa-mir-31,hsa-mir-489, |
| LINC00028 | hsa-mir-216a, |
| LGALS8-AS1 | hsa-mir-122, |
| KIRREL3-AS1 | hsa-mir-338, |
| KIAA0087 | hsa-mir-217, hsa-mir-338, hsa-mir-375, |
| KCNH1-IT1 | hsa-mir-106a, |
| ITPKB-IT1 | hsa-mir-551a, hsa-mir-31, hsa-mir-375, |
| ITGB5-AS1 | hsa-mir-137, |
| IGF2-AS | hsa-mir-17, hsa-mir-122, hsa-mir-338, |
| IDI2-AS1 | hsa-mir-217, hsa-mir-338, |
| hsa-mir-338 | NOVA1, |
| hsa-mir-31 | FOXD4L5, FOXD4L4, ZC3H12C, |
| hsa-mir-217 | DACH1, EZH2, TNFRSF21, NR4A2, |
| hsa-mir-216a | OXGR1, |
| hsa-mir-17 | TP53INP1,CCND1,ORMDL3,PARD6B,ELAVL2,CYBRD1,MAP3K12,RAB11FIP1,TNFRSF21,TGB8, SPOPL,ZNF280B,ZC3H12C,RABEP1,BTG3,ZBTB18,LIMA1,ENPP5,GINS4,CADM2,FOXQ1,PFKP,  TMEM123,E2F2,RUNDC1,SALL3,NRIP3,RUNX3,SERF1A,POLR3G,DUSP2,  RRAGD,ABHD2,FRS2,SMOC1,C9orf40, |
| hsa-mir-137 | KIT,FMNL2,PTGS2,YBX1, |
| hsa-mir-122 | AKT3, DUSP2, |
| hsa-mir-106a | TP53INP1,TNFRSF21,NRIP3,ZC3H12C,ZBTB18,RRAGD,SALL3,DUSP2,ELAVL2,ENPP5,BTG3,RUNX3,  RAB11FIP1,LIMA1,CADM2,FRS2,PFKP,SMOC1,CCND1,FOXQ1, |
| HPYR1 | hsa-mir-338, |
| HOTAIRM1 | hsa-mir-137,hsa-mir-17,hsa-mir-216a,hsa-mir-122,hsa-mir-338, |
| HAS2-AS1 | hsa-mir-137, |
| HAR1A | hsa-mir-122, |
| GRIK1-AS1 | hsa-mir-338, hsa-mir-375, |
| GLIS3-AS1 | hsa-mir-338, |
| FRY-AS1 | hsa-mir-301b,hsa-mir-122,hsa-mir-338,hsa-mir-375, |
| FOXP1-IT1 | hsa-mir-106a,hsa-mir-184,hsa-mir-338,hsa-mir-375, |
| ERVMER61-1 | hsa-mir-338, |
| ERVH48-1 | hsa-mir-301b,hsa-mir-137,hsa-mir-184,hsa-mir-338, |
| EGOT | hsa-mir-375, |
| EFCAB6-AS1 | hsa-mir-137, |
| DSCR9 | hsa-mir-106a,hsa-mir-184,hsa-mir-122,hsa-mir-338,hsa-mir-375, |
| DSCAM-AS1 | hsa-mir-137,hsa-mir-122,hsa-mir-338, |
| DNMBP-AS1 | hsa-mir-106a,hsa-mir-137,hsa-mir-17,hsa-mir-217,hsa-mir-122, |
| DNM1P35 | hsa-mir-122, |
| DLX6-AS1 | hsa-mir-106a,hsa-mir-17,hsa-mir-216a,hsa-mir-122,hsa-mir-31,hsa-mir-338, |
| DENND5B-AS1 | hsa-mir-301b, hsa-mir-17, |
| CYP1B1-AS1 | hsa-mir-301b,hsa-mir-137,hsa-mir-216a,hsa-mir-338, |
| CLRN1-AS1 | hsa-mir-137,hsa-mir-216a,hsa-mir-217,hsa-mir-338,hsa-mir-489, |
| CLDN10-AS1 | hsa-mir-137, |
| CHODL-AS1 | hsa-mir-137,hsa-mir-216a,hsa-mir-489, |
| CADM2-AS1 | hsa-mir-301b, |
| CACNA1C-AS1 | hsa-mir-551a, hsa-mir-217, hsa-mir-31, |
| C9orf170 | hsa-mir-216a, hsa-mir-217, hsa-mir-338, |
| C9orf106 | hsa-mir-184, hsa-mir-338, |
| C8orf49 | hsa-mir-301b,hsa-mir-106a,hsa-mir-17,hsa-mir-184,hsa-mir-216a,hsa-mir-122,hsa-mir-338,  hsa-mir-375, |
| C8orf31 | hsa-mir-106a, hsa-mir-122, hsa-mir-338, hsa-mir-375, |
| C7orf65 | hsa-mir-551a,hsa-mir-106a,hsa-mir-17,hsa-mir-216a,hsa-mir-31,hsa-mir-338,hsa-mir-489, |
| C5orf64 | hsa-mir-17,hsa-mir-184,hsa-mir-122,hsa-mir-31,hsa-mir-338,hsa-mir-375, |
| C2orf48 | hsa-mir-106a,hsa-mir-17,hsa-mir-216a,hsa-mir-122,hsa-mir-338, |
| C21orf91-OT1 | hsa-mir-31, |
| C1orf220 | hsa-mir-301b,hsa-mir-106a,hsa-mir-17,hsa-mir-216a,hsa-mir-122,hsa-mir-338, |
| C1orf143 | hsa-mir-17, hsa-mir-338, |
| C1orf132 | hsa-mir-106a,hsa-mir-17,hsa-mir-217,hsa-mir-375, |
| C12orf77 | hsa-mir-106a, hsa-mir-137, hsa-mir-17, hsa-mir-31, |
| C11orf72 | hsa-mir-106a,hsa-mir-137,hsa-mir-216a,hsa-mir-31,hsa-mir-375, |
| C11orf44 | hsa-mir-338, hsa-mir-375, hsa-mir-489, |
| C10orf91 | hsa-mir-106a, hsa-mir-122, hsa-mir-338, |
| C10orf111 | hsa-mir-137, hsa-mir-184, hsa-mir-217, hsa-mir-122, |
| BOK-AS1 | hsa-mir-184, hsa-mir-216a, |
| BACH1-AS1 | hsa-mir-301b, hsa-mir-137, |
| ATXN8OS | hsa-mir-122, hsa-mir-375, |
| ATP13A5-AS1 | hsa-mir-375, |
| ARHGEF26-AS1 | hsa-mir-106a, hsa-mir-17, hsa-mir-217, hsa-mir-489, |
| ARHGAP31-AS1 | hsa-mir-137, hsa-mir-122, |
| AP002478.1 | hsa-mir-551a,hsa-mir-106a,hsa-mir-17,hsa-mir-184,hsa-mir-216a,hsa-mir-122,hsa-mir-338,  hsa-mir-489, |
| AP001496.1 | hsa-mir-217, |
| AP001208.1 | hsa-mir-122, |
| AL589642.1 | hsa-mir-106a,hsa-mir-216a,hsa-mir-122,hsa-mir-31,hsa-mir-338,hsa-mir-489, |
| AL391421.1 | hsa-mir-137, hsa-mir-17, hsa-mir-338, |
| AL391001.1 | hsa-mir-122, hsa-mir-31, |
| AL359644.1 | hsa-mir-301b, |
| AL162430.2 | hsa-mir-216a, |
| AL158206.1 | hsa-mir-106a, |
| AL158151.2 | hsa-mir-122, |
| AL157387.1 | hsa-mir-375, |
| AL139002.1 | hsa-mir-301b, |
| AL137798.1 | hsa-mir-122, |
| AL136307.1 | hsa-mir-301b, |
| AL021395.1 | hsa-mir-122, |
| AL021068.1 | hsa-mir-217, hsa-mir-122, |
| AGAP11 | hsa-mir-551a,hsa-mir-106a,hsa-mir-17,hsa-mir-217,hsa-mir-375, |
| ADARB2-AS1 | hsa-mir-338, |
| AC124248.1 | hsa-mir-301b, hsa-mir-338, |
| AC104472.1 | hsa-mir-137, hsa-mir-31, hsa-mir-489, |
| AC103810.2 | hsa-mir-106a, hsa-mir-17, hsa-mir-338, |
| AC097717.1 | hsa-mir-301b, |
| AC093510.2 | hsa-mir-106a, hsa-mir-122, hsa-mir-375, |
| AC087269.1 | hsa-mir-31, hsa-mir-375, |
| AC083805.1 | hsa-mir-122, |
| AC080129.1 | hsa-mir-122, |
| AC073342.1 | hsa-mir-122, |
| AC068643.1 | hsa-mir-216a, |
| AC063962.1 | hsa-mir-17, |
| AC061975.7 | hsa-mir-106a, hsa-mir-17, |
| AC061975.6 | hsa-mir-106a, hsa-mir-17, |
| AC040173.1 | hsa-mir-17, hsa-mir-217, |
| AC027307.1 | hsa-mir-338, |
| AC025287.2 | hsa-mir-217, hsa-mir-122, |
| AC022148.1 | hsa-mir-106a, hsa-mir-17, hsa-mir-31, |
| AC022098.2 | hsa-mir-338, |
| AC021066.1 | hsa-mir-31, hsa-mir-338, hsa-mir-375, |
| AC020663.1 | hsa-mir-216a, hsa-mir-122, |
| AC016757.1 | hsa-mir-217, |
| AC015987.1 | hsa-mir-301b, hsa-mir-375, |
| AC012150.1 | hsa-mir-301b, |
| AC012074.1 | hsa-mir-122, |
| AC011442.1 | hsa-mir-122, |
| AC011374.1 | hsa-mir-106a, |
| AC009065.1 | hsa-mir-106a, |
| AC006305.1 | hsa-mir-137,hsa-mir-17,hsa-mir-216a,hsa-mir-338,hsa-mir-375, |
| AC005696.1 | hsa-mir-301b, hsa-mir-122, |
| AC005609.1 | hsa-mir-106a,hsa-mir-17,hsa-mir-122,hsa-mir-31,hsa-mir-338, |
| AC005544.1 | hsa-mir-31, hsa-mir-375, hsa-mir-489, |
| AC005035.1 | hsa-mir-301b, hsa-mir-375, |
| AC004917.1 | hsa-mir-17, |
| AC004832.1 | hsa-mir-17, hsa-mir-31, |
