# Supplementary figures and images for: lncRNA OSTN-AS1 May Represent a Novel Immune-Related Prognostic Marker for Triple-Negative Breast Cancer Based on Integrated Analysis of a ceRNA Network
Source: Front Genet. 2019 Sep 13;10:850. doi: 10.3389/fgene.2019.00850 (PMC6753250; doi:10.3389/fgene.2019.00850)

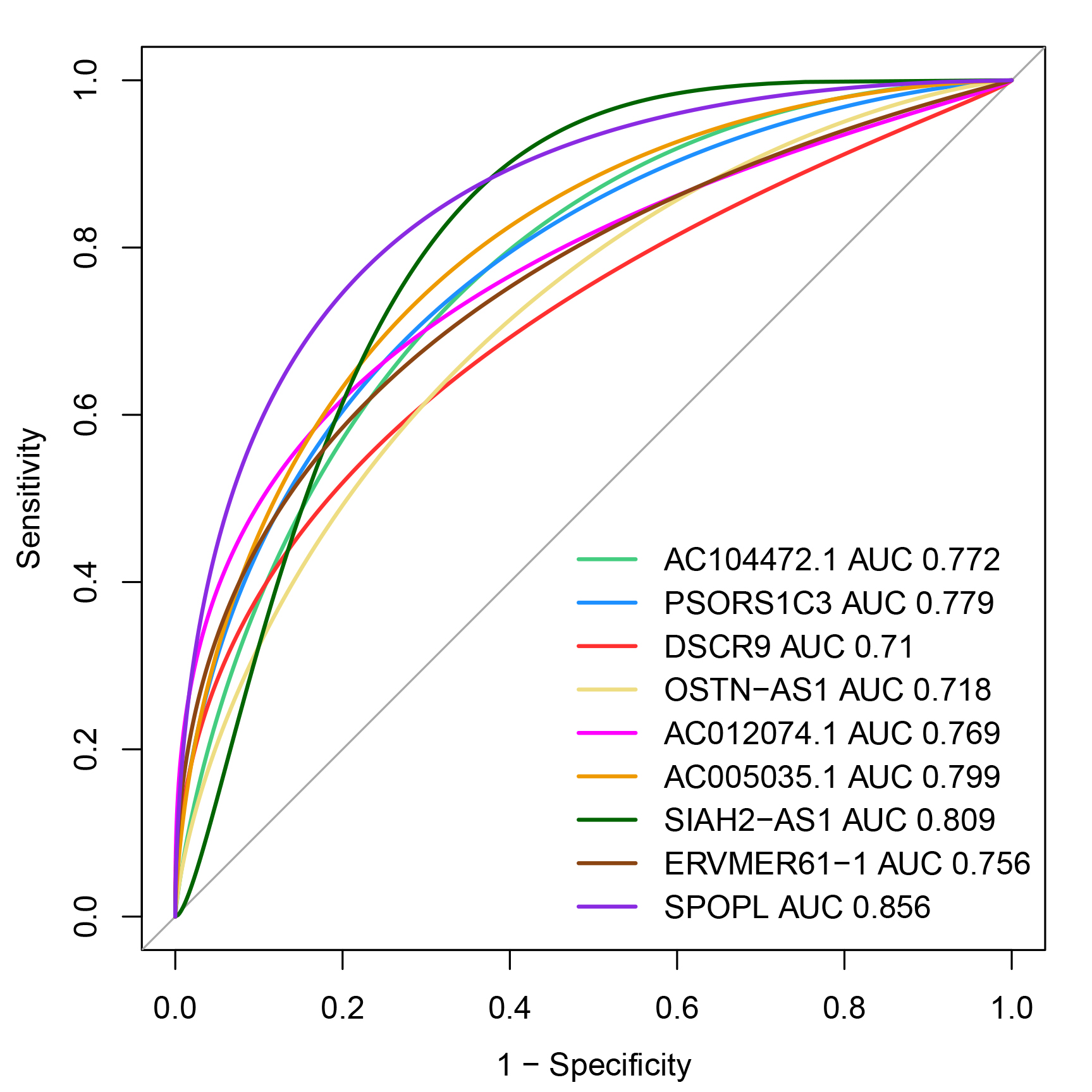

Supplement: Supplementary file 4 [file Image_1.jpeg]

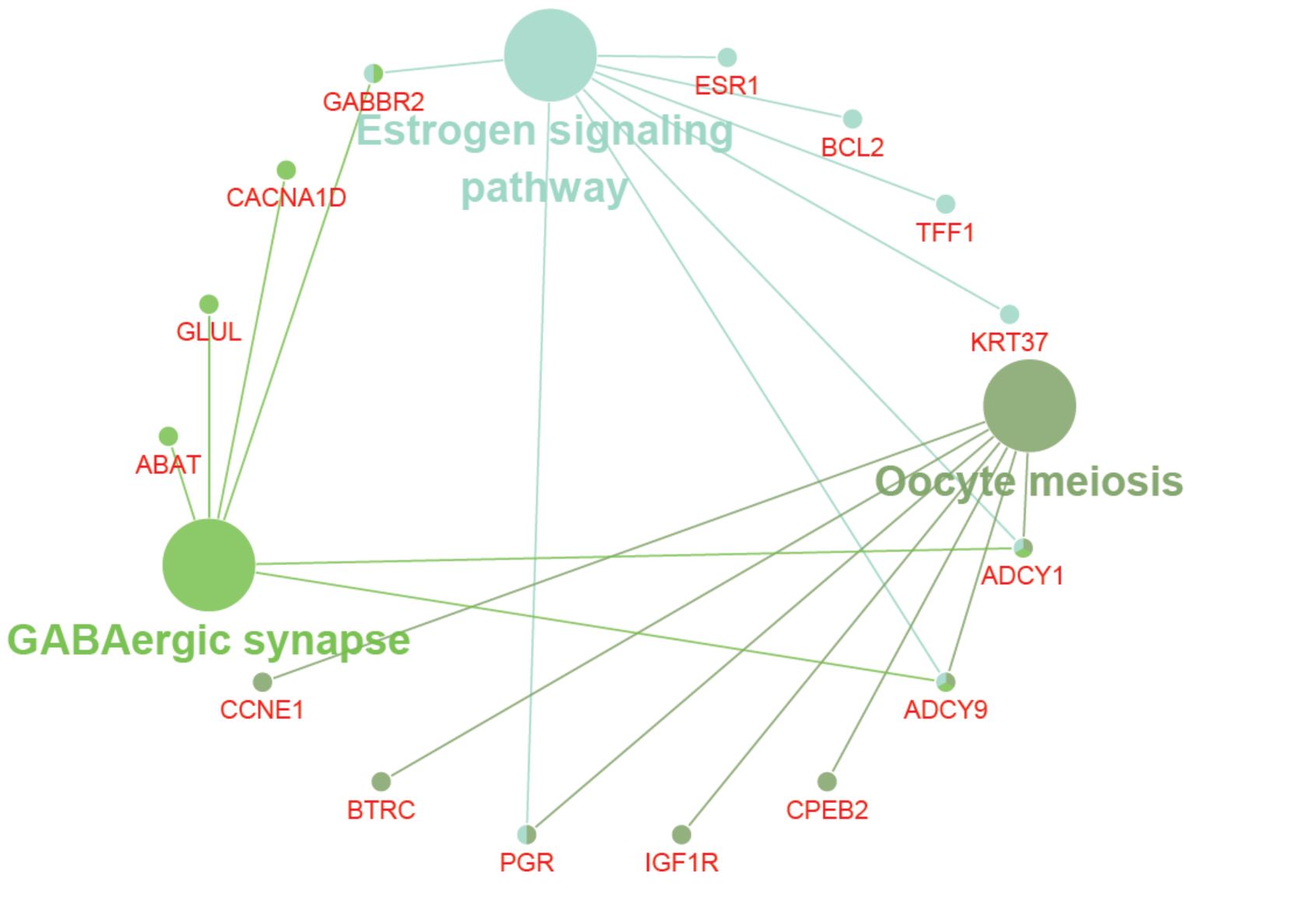

Supplement: Supplementary file 5 [file Image_2.jpeg]
